# Epistatic Association Mapping in Homozygous Crop Cultivars

**DOI:** 10.1371/journal.pone.0017773

**Published:** 2011-03-15

**Authors:** Hai-Yan Lü, Xiao-Fen Liu, Shi-Ping Wei, Yuan-Ming Zhang

**Affiliations:** 1 Section on Statistical Genomics, State Key Laboratory of Crop Genetics and Germplasm Enhancement, Nanjing Agricultural University, Nanjing, Jiangsu, China; 2 College of Information and Management Science, Henan Agricultural University, Zhengzhou, Henan, China; University of Massachusetts Amherst, United States of America

## Abstract

The genetic dissection of complex traits plays a crucial role in crop breeding. However, genetic analysis and crop breeding have heretofore been performed separately. In this study, we designed a new approach that integrates epistatic association analysis in crop cultivars with breeding by design. First, we proposed an epistatic association mapping (EAM) approach in homozygous crop cultivars. The phenotypic values of complex traits, along with molecular marker information, were used to perform EAM. In our EAM, all the main-effect quantitative trait loci (QTLs), environmental effects, QTL-by-environment interactions and QTL-by-QTL interactions were included in a full model and estimated by empirical Bayes approach. A series of Monte Carlo simulations was performed to confirm the reliability of the new method. Next, the information from all detected QTLs was used to mine novel alleles for each locus and to design elite cross combination. Finally, the new approach was adopted to dissect the genetic basis of seed length in 215 soybean cultivars obtained, by stratified random sampling, from 6 geographic ecotypes in China. As a result, 19 main-effect QTLs and 3 epistatic QTLs were identified, more than 10 novel alleles were mined and 3 elite parental combinations, such as Daqingdou and Zhengzhou790034, were predicted.

## Introduction

Germplasm resources play crucial roles in genetics, evolution and breeding, by forming the physical foundation of the study of genetic diversity [1]–[3], fueling much evolutionary research [4]–[6] and providing the raw material for breeders to produce new cultivars or to further improve the existing ones, due to the existence of many valuable genes in genetic resources [7]–[9]. The identification of valuable genes and markers associated with traits of interest will greatly increase the efficiency of plant breeding programs. However, these beneficial genes are largely unexplored due to the lack of appropriate statistical techniques. Meanwhile, as the complexity of the trait increase, breeding problems increase, for example, favorable alleles in exotic genetic resources are in unadapated genetic backgrounds and linked to other unfavorable alleles. This means that methods to utilize these favorable alleles in crop breeding also need to be further addressed. Accordingly, there is a critical need for in-depth study of methodologies for mining elite alleles in germplasm resources and for the utilization of these elite alleles in crop breeding.

During the past several decades, many attempts have been made to mine elite alleles for objective traits of interest. In early studies, many genes for qualitative traits in crop breeding were studied with morphological and biochemical approaches [10]–[13], and those for complex diseases in human genetics were identified by both sibling pair analysis [14]–[18] and pedigree analysis [19]–[21]. The introduction of molecular markers has facilitated the genetic association analysis of complex diseases in humans, animals and plants. Single-marker association analysis [22] and, later, genome-wide association study (GWAS) have been widely used in human genetics [23]. There has been substantial research of two aspects of GWAS: population structure [24]–[29] and mixed genetic models [30]–[32]. However, only one QTL was analyzed at a time in the above models. Likewise, although epistasis association analysis has been utilized in human genetics [33]–[37], all of the main genetic effects and gene interaction effects have not been simultaneously included in one genetic model. A full genetic model, including all the main and epistatic effects, could improve the power of QTL detection [38]–[41]. Several parameter estimation approaches such as LASSO [41], [42], empirical Bayes [43], and penalized maximum likelihood [38], [40] make this full genetic model possible. Therefore, epistasis association analysis with a full genetic model is feasible in crop germplasm resources.

In the past, most crop breeding methods were based on selection for observable phenotypes and breeding efficiency without markers is simply a function of heritability and choice of parental material. To date molecular markers have improved efficiency of selection largely for traits under simple genetic control and in specific conditions where marker selection is easier/cheaper than phenotypic selection [44]–[50]. However, this approach is only feasible for the improvement of one or several independent genes. If there are interactions among the objective genes, breeding strategy must be addressed by the incorporation of the epistasis [51], [52]. Carlborg and Haley [53] showed that epistasis is a common response to selection in breeding programs. Therefore, genetic interaction should be considered in crop breeding strategies.

One purpose of the genetic analysis of quantitative traits is to design a suitable breeding strategy, called breeding by design [54]. However, genetic analysis and crop breeding have traditionally been performed separately; for example, most genetic analyses exclusively use biparental crosses, but these are rarely used alone in commercial breeding. Therefore, the results of these biparental cross experiments have limited roles in breeding practice [55]–[57]. However, direct mapping of QTLs in natural populations, such as crop cultivars, is both economical and practical because the population being mapped is readily available, and the identified QTLs are directly applicable [31].

The purpose of this study was to develop an epistatic association mapping (EAM) approach in homozygous crop cultivars. We described detailed genetic and statistical models of epistasis association analysis in crop cultivars. All the parameters were estimated using the empirical Bayes approach. Our methods were confirmed by real data analysis in soybean and by a series of Monte Carlo simulation experiments.

## Results

### Phenotypic variation

**Figure 1:**
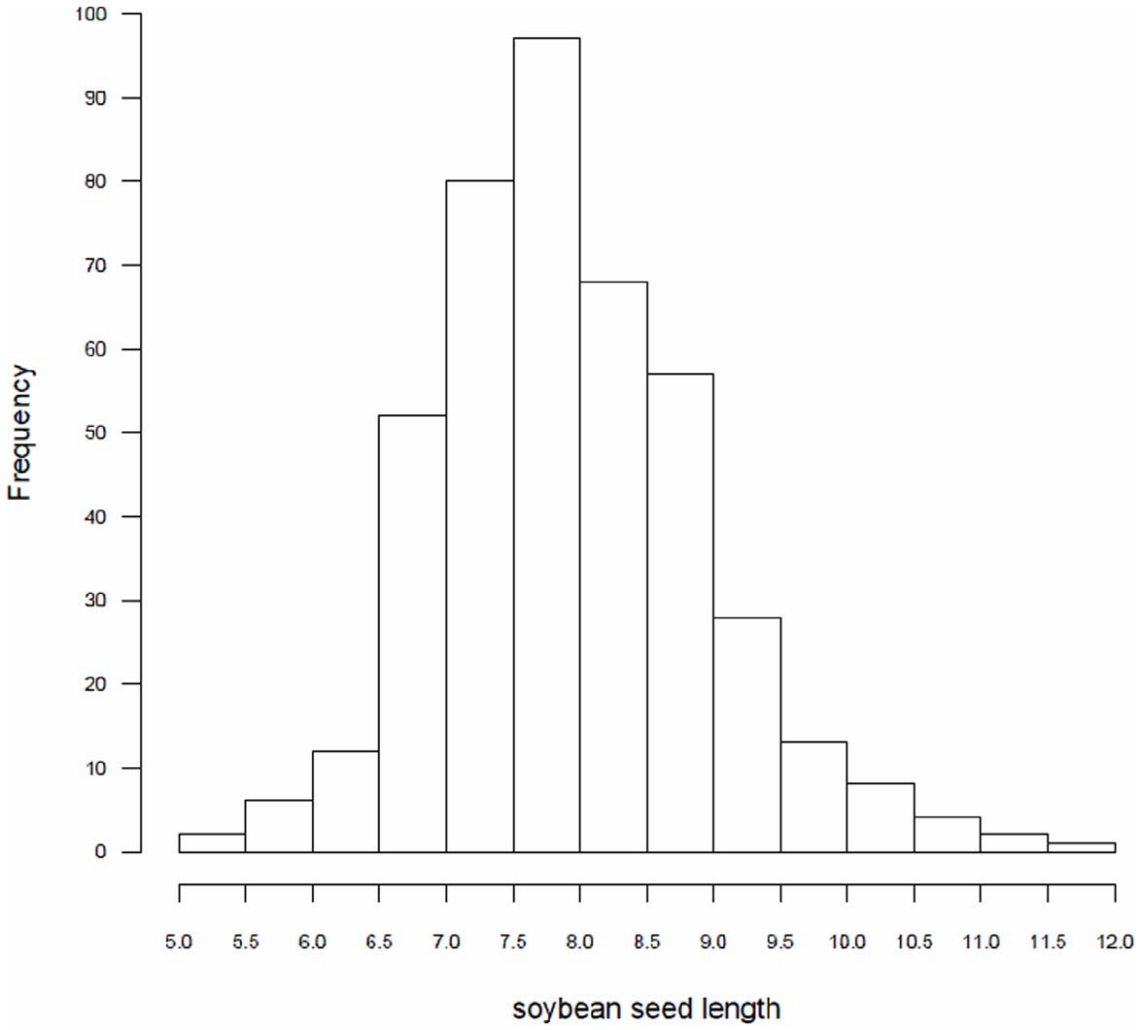
Frequency distribution for soybean seed length.

We measured seed length in 215 soybean cultivars. The minimum, maximum, average, median, standard deviation, coefficient of variation, skewness and kurtosis values were 5.30, 11.85, 7.94, 7.86, 0.99, 12.43, 0.61 and 0.91, respectively. Results from ANOVA showed that there is significant difference among cultivars (P<10^−4^) and there are no significant differences between years (P = 0.192) and among cultivar × year interactions (P = 0.328). This means that in the cultivar population, there is a large amount of genetic variation, which exhibits a continuous normal distribution (**Fig. 1**).

### Epistasis association mapping

Two years of phenotypic observations, along with information on 134 SSR molecular markers, were used to dissect the genetic basis of seed length in soybean. In the full model, 9,180 effects needed to be estimated, 40 times larger than the sample size. We adopted a two-stage method [58]. Nineteen main-effect QTLs and 3 epistatic QTLs for seed length in soybean were detected by EAM (Table 1). All of these QTLs were nearly evenly distributed along the soybean genome, except for chromosomes H, J and L. Among these QTLs, the proportion of the total phenotypic variance was from 0.25% to 10.44% for main-effect QTLs and from 5.08% to 7.38% for epistatic QTLs, and each of 12 QTLs contributed greater than 5.0% of the variance. In addition, five loci were involved in epistatic interactions, and only one of these five (sat_342) had a significant main effect. This lack of main effects may create difficulties in detecting epistasis with other methods.

**Table 1:** Detected QTL for seed length in soybean cultivar population.

QTL	New method						Genome-wide association study		
	Chr.	Marker associated	Position (cM)	Variance *	LOD	*r* ^2^ (%)	F	P-value	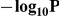
Main-effect	A1	satt382	26.42	0.1155	4.65	6.24	4.31	3.96E-7	6.40**
	A2	satt329	110.94	0.0199	2.53	1.08	7.10	1.53E-5	4.81
	B1	satt509	32.51	0.0426	7.89	2.30	4.67	3.69E-4	3.43
	B2	sat_342	20.31	0.0246	4.81	1.33	2.28	8.35E-3	2.08
	B2	satt534	87.59	0.1934	2.65	10.44	3.05	3.16E-5	4.50
	C2	sat_252	127.00	0.0962	4.89	5.19	3.73	1.99E-6	5.70
	D1b	sat_254	46.92	0.0709	4.12	3.83	4.24	1.27E-7	6.90**
	D1b	satt274	116.35	0.0083	6.93	0.45	10.97	2.27E-5	4.64
	D2	satt514	85.69	0.1059	6.33	5.72	2.81	1.31E-5	4.88
	D2	sat_365	87.39	0.1232	15.23	6.65	3.08	1.78E-6	5.74
	E	satt263	45.40	0.0592	5.67	3.20	3.71	1.17E-2	1.93
	F	satt656	135.12	0.1007	4.71	5.44	2.47	2.29E-3	2.64
	G	satt352	50.53	0.1307	5.37	7.06	1.74	3.46E-2	1.46
	G	AF162283	87.94	0.0222	3.77	1.20	6.38	1.86E-3	2.73
	I	sat_419	98.11	0.0047	6.24	0.25	7.64	2.98E-6	5.22
	K	satt441	46.20	0.0925	6.59	5.00	5.22	1.04E-7	6.98**
	M	sat_256	74.53	0.0893	2.56	4.82	2.57	5.01E-3	2.30
	N	satt022	102.06	0.1113	11.99	6.01	2.16	2.92E-3	2.53
	O	sat_274	107.58	0.0446	2.64	2.41	2.61	5.21E-4	3.28
Epistasis	B2 & C1	sat_342 & AW277661	20.31 & 74.79	0.1367	7.71	7.38	4.04	6.72E-6	5.17
	D1a & E	sat_160 & satt411	104.28 & 12.92	0.0941	3.06	5.08	3.74	3.07E-4	3.51
	D1b & E	satt459 & satt411	118.62 & 12.92	0.1224	5.61	6.61	6.73	1.33E-3	2.88

*: Calculated by 
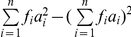
 for main-effect QTL and 
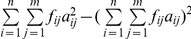
 for epistatic QTL, where *f* is allelic frequency, *a* is allelic effect and *n* and *m* is the number of alleles at the *i*th and *j*th loci. The same is true for the later tables.

**: QTL identified by genome-wide association study with the critical value at the 0.05 level of significance determined by 1000 permutation experiments.

**Figure 2:**
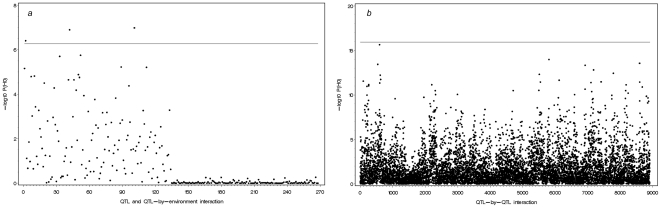
The 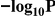 score profile of the soybean genome scan in the genome-wide association study for seed length in soybean. (a) Main-effect QTL and QTL-by-environmental interaction, and (b) QTL-by-QTL interaction. The critical values at the 0.05 level of significance, indicated by horizontal line, were determined by 1000 permutation experiments.

To compare the proposed approach with regular genome-wide association study (GWAS), the GWAS was used to analyze the above dataset. Results showed that three main-effect QTL, linked with markers satt382, sat_254 and satt441, respectively, were detected (**Fig. 2a**) and no significant environmental and epistatic interactions were identified (**Fig. 2**). These results are similar to those by the proposed approach in two aspects. First, the three main-effect QTLs detected by the GWAS are also identified by the proposed method. Second, no significant environmental interaction is detected by the above two approaches. However, there are some differences as well. The main difference is that the new approach can detect more main-effect and epistatic QTLs than the GWAS.

### Mining elite alleles

**Figure 3:**
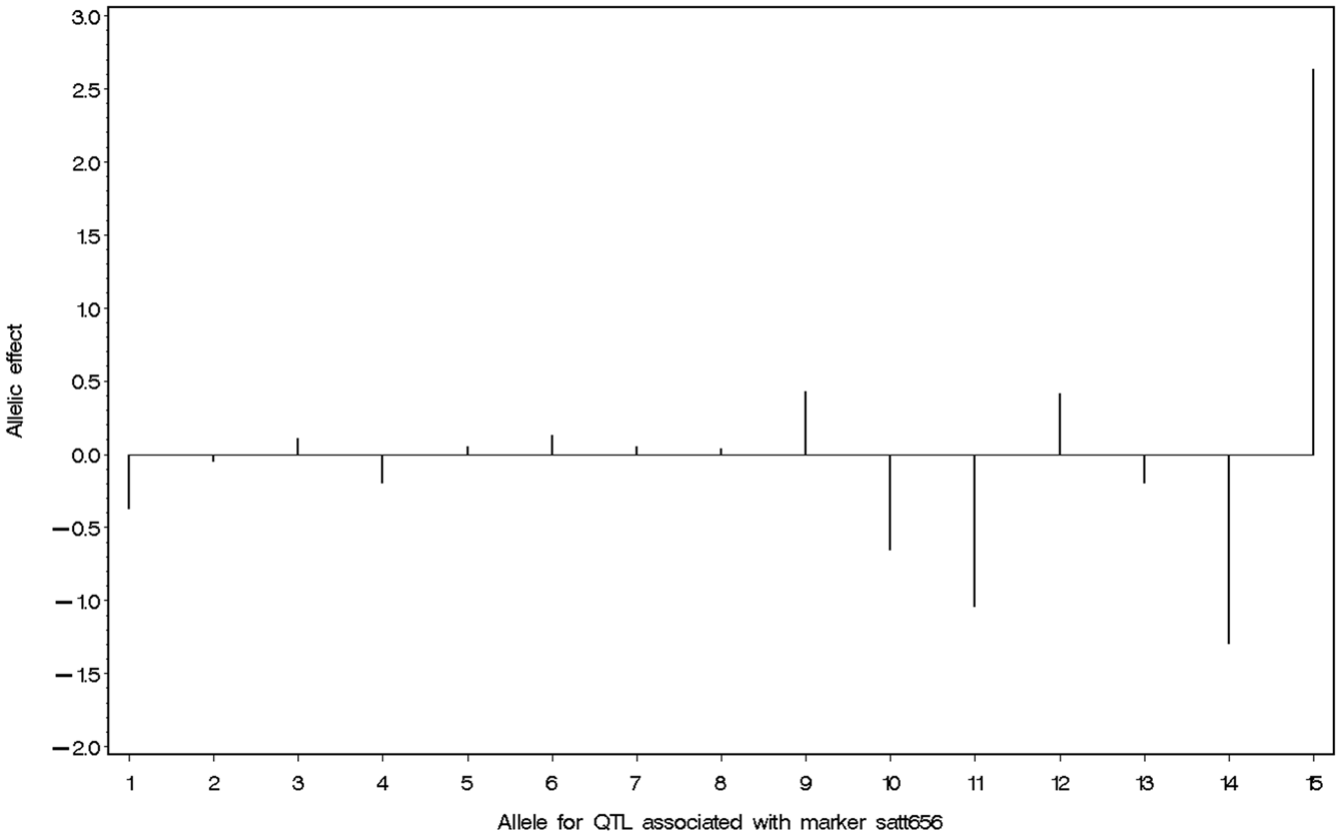
Allelic effects for QTL associated with marker satt656 for soybean seed length (mm).

The allelic effects of the cultivars were evaluated for all the identified loci for soybean seed length. The reduced model that includes the total mean, the population structure, all the identified loci and the residual error was a mixed model equation. In the reduced model, the allelic effects at each locus were estimated by a maximum likelihood approach. If we want to increase the trait value, we should take the allele with the largest positive effect per main-effect QTL as novel allele. If decreasing the trait value is our selection objective, we should take the allele with the largest negative effect per main-effect QTL as novel allele. The same is true for allele combination of epistatic QTL. The summary statistics for novel allele or allele combination are given in Table 2. These results show that there is one novel allele for each main-effect locus or one novel allele combination for each epistatic QTL. For example, for the locus linked to marker satt656, all the allelic effects are showed in **Fig. 3**, and novel allele is the allele with an effect of 2.63. Similarly, for the interaction between markers sat_342 and AW277661, novel allele combination is the allele combination with an effect of 1.29. The novel allele and allele combination were found in the Zhengzhou 790034 and Guangxibayuehuang cultivars, respectively.

**Table 2:** The information of novel allele for QTL with *r* ^2^ larger than 5%.

QTL	Chr.	Marker associated	Position (cM)	Novel allele (bp)	Effect (mm)	Cultivar withnovel allele
Main-effect	A1	satt382	26.42	295	0.64	Qinyan 1
	B2	satt534	87.59	185	1.22	Zhenghezhibanzi
	C2	sat_252	127.00	276	1.00	Taixinghanludou
	D2	satt514	85.69	242	1.11	Caishengzi
	D2	sat_365	87.39	286	0.95	Dandou 2
	F	satt656	135.12	182 or 170	2.63	Zhengzhou 790034
	G	satt352	50.53	178	0.87	Ya'anguanhualiyuebao
	K	satt441	46.20	282	1.11	Nannongdahuangdou
	N	satt022	102.06	277	0.94	Dandongdaliqing
Epsitasis	B2 & C1	sat_342 & AW277661	20.31 & 74.79	288 & 301	1.29	Guangxibayuehuang
	D1a & E	sat_160 & satt411	104.28 & 12.92	190 & 109	0.99	Anbaishuidou
	D1b & E	satt459 & satt411	118.62 & 12.92	195 or 189 & 106	1.09	Zhengzhou 74064

### Predictions for elite cross combination

The elite cross combinations could be predicted from all the detected loci and their effects by using the method described below. In a hypothetical cross between two cultivars, all types of RILs would be produced. In these RILs, seed length could be predicted by the combined effects of all the detected loci. The best RIL with maximum seed length in one cross would represent the cross. The best cross with maximum seed length in all the crosses could be selected by comparing all the crosses. In this study, the best three crosses were Daqingdou × Zhengzhou790034, Zhenghe- zhibanzi × Zhengzhou790034, and Liyangdawuhuangdou × Zhengzhou 790034. The presence of Zhengzhou790034 in the three best crosses indicated that it contained the best allele or allele combination.

### Monte Carlo simulation studies

#### Evaluation of the performance of the proposed approach

The first simulation experiment was designed to investigate the effect of QTL heritability on QTL mapping in crop cultivars. The results show that the precision and power of the detection of QTLs increase with increasing QTL heritability, and that the false positive rate (FPR) is only 0.0244% (Table S2).

In the second simulation experiment, we investigated the effect of sample size by randomly sampling 100, 200, or 300 non-founder lines. The other parameters were the same as those in the first simulation experiment. As expected, the precision and power increased with increasing sample size (Table S3). Sample sizes under 300 yield much better results than those under 200; we recommend a sample size of 300 for future studies.

The third simulation experiment compared the effect of the number of alleles on QTL mapping in crop cultivars. We set the numbers of alleles at 2, 3 and 4; other parameters were the same as those in the first simulation experiment. The results showed that precision and power decrease as the number of alleles increases (Table S4). The results also imply that the SNP or indel markers are better than the other markers.

In the fourth simulation experiment, the effect of allelic frequency on QTL mapping was assessed by setting the frequency ratio of the two alleles as 1∶1 (uniform distribution), 1∶2 (skewed distribution) or 1∶3 (skewed distribution). The other parameters were the same as those in the first simulation experiment. The results showed that skewed distribution decreased the statistical power (Table S5), indicating that rare alleles should be preferentially studied in association analyses.

#### The detection of QTL-by-environment interaction

To investigate whether environmental effects could be detected, all the cultivars were evaluated in multiple environments. In the fifth simulation experiment, two environments, ten main-effect QTL and five QTL-by-environment interactions were simulated. The new method holds greater power for detecting QTL-by-environment interactions than for the main-effect QTL, and the FPR is lower than 0.06% (Table 3). To further demonstrate the performance of the new method, in the sixth simulation experiment, we designed a large genome with high density markers. In total, 510 markers were simulated on ten chromosome segments 1,000 cM long, with an average marker interval of 2 cM. The other parameters were the same as those in the fifth simulation experiment. The same trend in the fifth experiment was obtained (Table 4), indicating that our method works in large genomes with a high marker density.

**Table 3:** Environmental interaction detection in Monte Carlo simulation experiment (200 replicates).

QTL	True value				Estimate			
	Chr.	Position (cM)	Variance	*r* ^2^ (%)	Power (%)	Position (cM)	Variance	*r* ^2^ (%)
Main-effect	1	70.3	0.926	5.0	100.0	70.3(0.0)	0.8934(0.2176)	4.94(1.21)
		262.8	0.926	5.0	99.5	262.8(0.0)	0.8912(0.2131)	4.92(1.15)
	2	401.4	0.370	2.0	95.0	401.4(0.0)	0.3552(0.1366)	1.96(0.74)
		438.8	0.556	3.0	99.0	438.8(0.0)	0.5215(0.1589)	2.88(0.86)
	3	601.6	0.926	5.0	100.0	601.6(0.0)	0.8816(0.2125)	4.87(1.15)
	8	1653.4	0.185	1.0	58.0	1653.4(0.4)	0.2097(0.0858)	1.15(0.47)
		1747.6	0.370	2.0	93.5	1747.6(0.0)	0.3384(0.1372)	1.87(0.76)
	9	1944.7	1.852	10.0	100.0	1944.7(0.0)	1.8511(0.3121)	10.22(1.59)
	10	2145.2	0.926	5.0	100.0	2145.2(0.0)	0.9322(0.2352)	5.15(1.23)
		2181.6	0.926	5.0	100.0	2181.6(0.0)	0.9081(0.2051)	5.02(1.09)
Environment			0.926	5.0	96.0		0.8744(0.2580)	4.82(1.39)
Environmental	1	55.6	0.463	2.5	97.0	55.6(0.0)	0.4229(0.1391)	2.33(0.75)
interaction	2	401.4	0.463	2.5	98.0	401.4(0.0)	0.4465(0.1678)	2.46(0.88)
		438.8	0.926	5.0	100.0	438.8(0.0)	0.8867(0.2100)	4.90 (1.12)
	3	682.7	0.926	5.0	100.0	682.7(0.0)	0.9016(0.2190)	4.98(1.19)
	8	1747.6	1.852	10.0	100.0	1747.6(0.0)	1.8344(0.2903)	10.13(1.47)
False positive rate (%)		0.0550						

**Table 4:** Environmental interaction detection under the situations of large genome and high-density markers (200 replicates).

QTL	True value				Estimate			
	Chr.	Position (cM)	Variance	*r* ^2^ (%)	Power (%)	Position (cM)	Variance	*r* ^2^ (%)
Main-effect	1	40	0.926	5.0	99.5	40.0(0.0)	0.8889(0.2126)	4.92(1.15)
		60	0.926	5.0	100.0	60.0(0.0)	0.8813(0.2233)	4.88(1.21)
	2	120	0.370	2.0	93.0	120.0(0.0)	0.3579(0.1313)	1.98(0.73)
		160	0.556	3.0	97.0	160.0(0.0)	0.5166(0.1869)	2.85(1.01)
	3	254	0.926	5.0	100.0	254.0(0.0)	0.8938(0.2097)	4.93(1.07)
	5	430	0.185	1.0	63.0	430.0(0.0)	0.1984(0.0801)	1.10(0.45)
		460	0.370	2.0	93.0	460.0(0.0)	0.3570(0.1282)	1.98(0.73)
	7	656	1.852	10.0	100.0	656.0(0.0)	1.8482(0.3380)	10.23(1.81)
	9	842	0.926	5.0	100.0	842.0(0.0)	0.9066(0.2507)	5.02(1.38)
		852	0.926	5.0	99.5	852.0(0.0)	0.8996(0.2350)	4.97(1.24)
Environment			0.926	5.0	91.5		0.9654(0.3431)	5.30(1.79)
Environmental	1	58	0.463	2.5	96.5	58.0(0.1)	0.4351(0.1290)	2.41(0.73)
interaction	2	136	0.463	2.5	95.0	136.0(0.0)	0.4469(0.1554)	2.47(0.86)
	3	254	0.926	5.0	100.0	254.0(0.0)	0.8787(0.2201)	4.86(1.18)
	5	460	0.926	5.0	100.0	460.0(0.0)	0.8878(0.2214)	4.91(1.21)
	9	842	1.852	10.0	100.0	842.0(0.0)	1.7989(0.3053)	9.95(1.59)
False positive rate (%)		0.0597						

#### The identification of QTL-by-QTL interaction

To demonstrate whether QTL-by- QTL interactions could be detected, all epistatic effects between two main-effect QTLs were included in the full model. In the final simulation experiment, 50 markers were evenly distributed in five linkage groups 450 cM in length. Five main-effect QTLs, 3 QTL-by-environment interactions and 5 QTL-by-QTL interactions were simulated. The results (Table 5) show that the estimates for the positions and variances of simulated QTLs are close to their true values, and the power in the detection of QTL is high (e.g., over 80% for the QTLs with a heritability over 2%), especially for QTL-by-QTL interactions.

**Table 5:** Epistatic QTL detection in Monte Carlo simulation experiment (200 replicates).

QTL	True value				Estimate			
	Chr.	Position (cM)	Variance	*r* ^2^ (%)	Power(%)	Position (cM)	Variance	*r* ^2^ (%)
Main-effect	1	50	0.4	2	83.5	50.0(0.0)	0.3967(0.1317)	2.04(0.66)
	2	100	1.0	5	97.5	100.0(0.0)	0.9441(0.2544)	4.88(1.31)
	3	200	2.0	10	99.5	200.0(0.0)	1.9239(0.5039)	9.90(2.35)
	4	350	0.4	2	82.0	350.0(0.0)	0.3953(0.1371)	2.03(0.70)
	5	400	1.0	5	95.5	400.0(0.0)	0.9741(0.3574)	4.98(1.71)
Environment			1.0	5	99.0		0.9408(0.2294)	4.86(1.14)
Environmental	2	150	0.4	2	98.5	150.0(0.0)	0.3766(0.1255)	1.96(0.67)
interaction	3	270	2.0	10	100.0	270.0(0.0)	1.9703(0.3007)	10.21(1.57)
	5	400	1.0	5	99.5	400.0(0.0)	0.9354(0.2261)	4.83(1.12)
Epistasis	1 & 2	10 & 130	0.4	2	97.0	10.0(1.0) & 129.9(1.4)	0.3444(0.1262)	1.78(0.65)
	2 & 3	100 & 250	1.0	5	100.0	100.0(0.0) & 250.0(0.0)	0.9825(0.2196)	5.09(1.13)
	3 & 5	200 & 400	0.4	2	85.5	200.0(0.0) & 399.9(1.5)	0.3842(0.1275)	1.98(0.66)
	3 & 4	270 & 360	2.0	10	100.0	270.1(0.7) & 360.0(1.6)	1.9350(0.3605)	9.99(1.79)
	4 & 5	350 & 450	2.0	10	100.0	350.1(0.7) & 450.0(0.0)	1.9814(0.3912)	10.25(1.98)
False positive rate (%)			0.0545					

## Discussion

The approach proposed in this work has several advantages over the approaches of previous association analysis studies. First, main, environmental, QTL-by- environment and QTL-by-QTL interactions were simultaneously considered in our full genetic model, improving the statistical power [38]–[41]. Although multi-locus genetic models have been proposed in plant genetics [59]–[62], they have difficulty combining both QTL-by-environment and QTL-by-QTL interactions. Epistasis association mapping has been developed in human genetics [33]–[37], but here the epistasis was identified by two-dimensional scan, and significant effects in the two-dimensional scan were further tested in one genetic model. Second, epistasis association analysis was first integrated with crop breeding by design. In the past, the results from QTL mapping have had limited utility in breeding practice, due to the use of a simple cross population or the neglect of epistasis in the detection of QTLs. We designed an elite cross combination to take these two issues into account. Third, it is easy to extend the proposed approach to nested association analysis. The commonality is that all the individuals in the mapping populations are inbred lines. The difference is that the pedigree is general for the present study and relatively simple for nested association analysis. Therefore, the new method is suitable for nested association analysis and human genetics. Fourth, the FPR is minimized in the new method. A shrinkage estimation method, empirical Bayes (eBayes), was adopted to estimate all types of effects in the full model so that the FPR was less than 0.06%.

At present the most widely used genome-wide association study (GWAS) is analysis of variance or mixed model approaches with the control of false discovery rate. In theory, it is similar to single-marker analysis for main-effect QTL and two-marker analysis for epistatic QTL, and the difference is that the GWAS requires the setting of a significance threshold at the genome-wide level. However, it does not overcome the shortcomings of marker analysis. If a trait of interest is controlled by multiple QTLs, whether the QTL under consideration can be detected depends on the proportions of phenotypic variance explained by both this QTL and background QTLs. If the proportion by background QTLs is large, large residual variance will result in a decreased power in the detection of the current QTL and sometime the QTL can not be identified. In the new approach, this issue can be avoided, because a full model that includes all kinds of QTL in one genetic model results in a small residual variance. This explains why some main-effect QTLs and all the epistatic QTLs can not be mapped in the soybean genome-wide association study.

Prediction of elite cross combination is based on the assumption that dominance and dominance-type epistasis effects are absent. If the breeding objective is the development of inbred lines or cultivars as often the case in self-pollinated crops, the prediction may be useful. If these non-additive effects are important, then the prediction would not reliable. This issue needs to be addressed in the future.

Xu [41] described a linear model in which the dimensions of the genotypic value vector and its incidence matrix depend on the number of genotypes for the locus. In theory, this model matches the situation under study. However, the model dimensions will increase rapidly. Therefore, it is preferable to gather more samples or reduce the number of effects considered [38], [63] to reduce the dimensions of the model. In this study, we designed a special incidence matrix such that there is one variable for each main-effect QTL. Simulation studies show that this approach works well. If the number of markers is large, the number of effects in the model is enormous. In this case, the two-stage method of He and Zhang [58] is recommended. We adopted this approach in our analysis of real data, and the results were consistent with those of He and Zhang [58] and He et al. [64]. The new approach works well if the marker interval length is approximately 5 cM. However, one must delete some closely linked markers if the interval length is less than 5 cM [64].

We compared the QTLs of seed length in soybeans with the QTLs in previous studies. Although few common markers existed between their data and ours, some loci that we detected were also detected in previous studies. Seven QTLs linked to markers sat_342, satt534, satt514, sat_365, sat_254, sat_419 and sat_274 in this study were detected by Xu et al. [65]; four QTLs associated with markers satt411, satt329, satt022 and AW277661 in this paper were identified by Salas et al. [66]; one QTL close to marker sat_256 was confirmed by Li et al. [67]; and one QTL next to marker satt514 was mapped by Liang et al. [68]. The above results further confirmed the feasibility of the approach proposed in this study.

## Materials and Methods

### Soybean samples

We recently assembled a soybean association panel with 215 cultivars provided by the National Center for Soybean Improvement, China. All the cultivars were obtained by stratified random sampling from six geographic ecotypes in China [69], planted in three-row plots in a completely randomized design and evaluated at the Jiangpu experimental station at Nanjing Agricultural University in 2008 and 2009. The plots were 1.5 m wide and 2 m long. Five individuals and 20 seeds in the middle row of each plot were randomly picked to measure seed length by digital vernier caliper. The measurements were averaged over 20 seeds, and the mean was used in this study.

Approximately 0.3 g of fresh leaves obtained in 2008 from each cultivar was used to extract genomic DNA using the cetyltrimethylammonium bromide method as described by Lipp et al. [70]. To screen for polymorphisms among all the cultivars, PCR was performed with 134 simple sequence repeat (SSR) primer pairs. The primer sequences were obtained from the soybean database Soybase (http://www.ncbi.nlm.nih.gov). PCR was performed as described by Xu et al. [65].

### Population structure

For the soybean data, the STRUCTURE program was used to investigate the population structures of all selected cultivars [26]. The number of subpopulations (*K*) was set from 2 to 10. In the Markov chain Monte Carlo (MCMC) Bayesian analysis for each *K*, the length of a Markov chain consisted of 110,000 sweeps. The first 10,000 sweeps (the burn-in period) were deleted, and thereafter, the chain was used to calculate the mean of log-likelihood. This process was repeated 20 times, and the total average for mean log-likelihood at fixed *K* was used. STRUCTURE analysis with 134 SSR molecular markers showed that the log-likelihood increased with the increase of the model parameter *K*, so a suitable number of *K* could not be determined. In this situation, using the ad hoc statistic 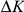, based on the rate of change in the log-probability of data between successive *K* values, STRUCTURE accurately detected the uppermost hierarchical level of structure [71]. Here, the 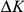 value was much higher for the model parameter 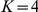 than for other values of *K*. By combining this high 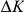 value with knowledge of the breeding history of these cultivars, we chose a value of 4 for *K*. The Q matrix was calculated based on SSR markers and incorporated into the mixed model of epistasis association analysis.

### Genetic model

The phenotypic value of a quantitative trait for the *i*th cultivar in the *j*th environment (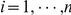;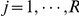), 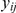, may be described by the following mixed model:
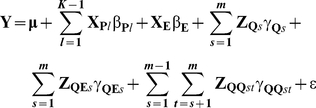
(1)where 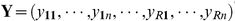; 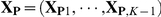 is the Q matrix for population structure; 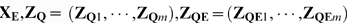 and 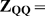 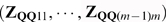 are the design matrices of the environment effect, main effect, QTL-by-environment interaction effect and QTL-by-QTL interaction effect, respectively; 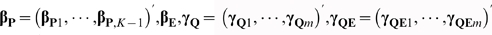 and 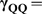 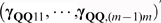 are the corresponding effects; and 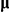 is the total average. The first three terms were viewed as fixed effects and the following three terms were considered random effects; therefore, model (1) was rewritten as
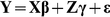
(2)where 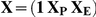, 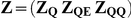, 
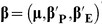
 and 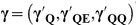.

### Parameter estimation

Several methods exist to simultaneously estimate the parameters in model (2); for example, eBayes [41], [43]. Here, we adopted eBayes. Briefly, the parameter vector in model (2) is 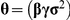. The priors and the likelihood are not described in detail here. The iteration process is given below.

The fixed effects were calculated by:
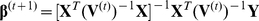
(3)

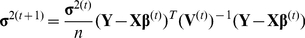
(4)where 
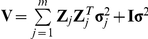
. Note that there is not an explicit solution for the estimation of 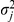, and it is updated by maximizing
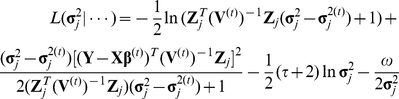
(5)where 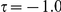 and 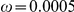.

The random effects, 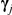, were predicted by best linear unbiased prediction (BLUP):
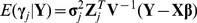
(6)


The posterior variance of 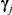 is
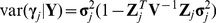
(7)


The proportion of phenotype variance explained by one random effect may be calculated by
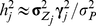
(8)


### Likelihood ratio test

The traditional likelihood ratio test (LRT), as described by Zhang and Xu [38], could not be performed in this study, due to an oversaturated epistatic genetic model. We proposed the following two-stage selection process to screen all the effects. In the first stage, all the effects with 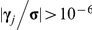 are picked up. In the second stage, the full model is modified so that only the effects that passed the first round of selection are included. Due to the smaller dimensionality of the reduced model, we can use the maximum likelihood method to reanalyze the data and perform the LRT. The procedure for the LRT is below.

The overall null hypothesis is no effect of the QTL at the locus of interest, denoted by 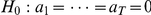, where 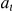 is the effect of the *t*th allele. If we solve the maximum likelihood estimation of the parameters under the restriction of 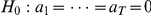 and calculate the log-likelihood value using the solutions with this restriction, we obtain 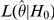. We can also evaluate the log-likelihood value of the solutions without restrictions and obtain 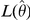. Therefore, the LR test statistic is
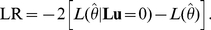
(9)


Other test statistics can be used in similar ways. The significance threshold of the LOD score was set at 2.5 for our real data analysis, where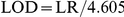.

### Genome-wide association study

First, phenotypic values for seed length in 215 soybean cultivars were corrected using population structure obtained by STRUCTURE software. Then, the corrected phenotypes along with SSR marker information were used to carry out genome-wide association studies for main-effect QTLs, environmental interactions and QTL-by- QTL interactions by ANOVA. Finally, critical values at the 0.05 level of significance were determined by 1000 permutation experiments and thus significant QTL could be identified.

### Simulation design

We performed seven simulation experiments in this study. In the first, the simulated pedigree was the maize pedigree described by Zhang et al. [31], [61]. The number of inbred lines within the maize pedigree was 404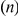. Of these, 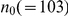 were base (founder) lines, which were in linkage equilibrium so that the genotypes for markers and QTLs with two alleles could be simulated. Non-founders (*n_1_* = 301) were bred via repeated self-pollination of a hybrid between two inbred lines. Thus, each non-founder line represents a recombinant inbred line (RIL) with respect to a known pair of parents. The genotypes of all the non-founders could be generated from the genotypes of their parents, analogous to simulating the genotypes of RILs from their parents. All of the non-founder lines could be used to detect QTLs. To mimic the actual linkage maps that did not have equally spaced markers, 153 markers were simulated on ten chromosome segments of length ∼2258.70 cM, with an average marker interval of 14.86 cM. A total of 20 QTLs, all of which overlapped with the markers, were simulated; the sizes and locations of the QTLs are listed in Table 3. The allelic effects were calculated by relating the genetic variance of the QTL to both the allelic frequencies and the allelic number. The phenotypic value of each line was the sum of the corresponding QTL genotypic values and the residual error, with an assumed normal distribution. Each simulation run consisted of 200 replicates. For each simulated QTL, we counted the samples in which the LOD statistic surpassed 3.0. The ratio of the number of such samples (*m*) to the total number of replicates (200) represented the empirical power of this QTL. The false-positive rate was calculated as the ratio of the number of false-positive effects to the total number of zero effects considered in the full model. The other simulation experiments were performed similarly. All simulated parameters are given in Table S1.

## Supporting Information

Table S1 **Simulated parameters in all the simulation experiments.**
(DOC)

Table S2 **Multi-QTL detection under various QTL heritabilities in the first simulation experiment (200 replicates).**
(DOC)

Table S3 **Effect of sample size on multi-QTL mapping in the second simulation experiment (200 replicates).**
(DOC)

Table S4 **Effect of the number of alleles on multi-QTL mapping in the third simulation experiment (200 replicates).**
(DOC)

Table S5 **Effect of allelic distribution on multi-QTL mapping in the fourth simulation experiment (200 replicates).**
(DOC)
