# Supplementary material for: Epistatic Association Mapping in Homozygous Crop Cultivars
Source: PLoS One. 2011 Mar 15;6(3):e17773. doi: 10.1371/journal.pone.0017773 (PMC3058038; doi:10.1371/journal.pone.0017773)
Supplement: Table S1 — Simulated parameters in all the simulation experiments. (DOC) [file pone.0017773.s001.doc]

**Table S1. Simulated parameters in all the simulation experiments**

| **Simulated**  **experiment** | **Pedigree** | | | **No. chr.** | **Marker**  **density**  **(cM)** | **Genome**  **length**  **(cM)** | **Allele of both QTL and marker** | |
| --- | --- | --- | --- | --- | --- | --- | --- | --- |
|  | **Name** | **No. of**  **founders** | **No. of**  **non-founders** |  |  |  | **Number** | **Distribution** |
| **1** | **Maize** | **103** | **301** | **10** | **Unequal** | **2258.7** | **2** | **1:1** |
| **2** | **Maize** | **103** | **100, 200, 300** | **10** | **Unequal** | **2258.7** | **2** | **1:1** |
| **3** | **Maize** | **103** | **301** | **10** | **Unequal** | **2258.7** | **2, 3, 4** | **1:1, 1:1:1, 1:1:1:1** |
| **4** | **Maize** | **103** | **301** | **10** | **Unequal** | **2258.7** | **2** | **1:1, 1:2, 1:3** |
| **5** | **Maize** | **103** | **301** | **10** | **Unequal** | **2258.7** | **2** | **1:1** |
| **6** | **Maize** | **103** | **301** | **10** | **Equal, 2** | **100×10** | **2** | **1:1** |
| **7** | **Maize** | **103** | **301** | **5** | **Equal, 10** | **90×5** | **2** | **1:1** |
