# Supplementary material for: Epistatic Association Mapping in Homozygous Crop Cultivars
Source: PLoS One. 2011 Mar 15;6(3):e17773. doi: 10.1371/journal.pone.0017773 (PMC3058038; doi:10.1371/journal.pone.0017773)
Supplement: Table S2 — Multi-QTL detection under various QTL heritabilities in the first simulation experiment (200 replicates). (DOC) [file pone.0017773.s002.doc]

**Table S2. Multi-QTL detection under various QTL heritabilities in the first simulation experiment (200 replicates)**

| **True value** | | | |  | **Estimate** | | | |
| --- | --- | --- | --- | --- | --- | --- | --- | --- |
| **Chr.** | **Position (cM)** | **Variance** | ***r*^2^ (%)** |  | **Power (%)** | **Position (cM)** | **Variance** | ***r*^2^ (%)** |
| **1** | **85.1** | **0.625** | **2.5** |  | **80.0** | **85.01(1.2)** | **0.6009(0.2453)** | **2.48(0.96)** |
|  | **222.6** | **1.875** | **7.5** |  | **98.5** | **222.6(0.0)** | **1.8072(0.4405)** | **7.49(1.82)** |
| **2** | **401.4** | **0.625** | **2.5** |  | **80.0** | **401.4(0.0)** | **0.6054(0.2336)** | **2.50(0.95)** |
|  | **438.8** | **1.875** | **7.5** |  | **99.0** | **438.8(0.0)** | **1.8154(0.4191)** | **7.51(1.68)** |
| **3** | **601.6** | **3.750** | **15.0** |  | **100.0** | **601.6(0.0)** | **3.7045(0.7234)** | **15.32(2.80)** |
| **8** | **1594.1** | **1.250** | **5.0** |  | **97.5** | **1594.1(0.0)** | **1.1960(0.3735)** | **4.94(1.51)** |
|  | **1653.8** | **1.250** | **5.0** |  | **90.5** | **1653.8(0.0)** | **1.1749(0.3308)** | **4.86(1.36)** |
| **9** | **1944.7** | **2.50** | **10.0** |  | **100.0** | **1944.7(0.0)** | **2.3862(0.5437)** | **9.87(2.14)** |
| **10** | **2119.6** | **2.50** | **10.0** |  | **99.5** | **2119.6(0.0)** | **2.3933(0.4972)** | **9.87(1.80)** |
|  | **2181.6** | **3.750** | **15.0** |  | **100.0** | **2181.6(0.0)** | **3.5902(0.6382)** | **14.85(2.47)** |
| **False positive rate (%)** | | **0.0244** | | | | | | |
