# Supplementary material for: Epistatic Association Mapping in Homozygous Crop Cultivars
Source: PLoS One. 2011 Mar 15;6(3):e17773. doi: 10.1371/journal.pone.0017773 (PMC3058038; doi:10.1371/journal.pone.0017773)
Supplement: Table S3 — Effect of sample size on multi-QTL mapping in the second simulation experiment (200 replicates). (DOC) [file pone.0017773.s003.doc]

**Table S3 Effect of sample size on multi-QTL mapping** in the second simulation experiment (200 replicates)

| **True value of QTL** | | | |  | **100** | | | |  | **200** | | | |  | **300** | | | |
| --- | --- | --- | --- | --- | --- | --- | --- | --- | --- | --- | --- | --- | --- | --- | --- | --- | --- | --- |
| **Chr.** | **Position**  **(cM)** | **Variance** | ***r*^2^ (%)** |  | **Power (%)** | **Position**  **(cM)** | **Variance** | ***r*^2^ (%)** |  | **Power (%)** | **Position**  **(cM)** | **Variance** | ***r*^2^ (%)** |  | **Power (%)** | **Position**  **(cM)** | **Variance** | ***r*^2^ (%)** |
| **1** | **85.1** | **0.625** | **2.5** |  | **7.0** | **83.0**  **(5.4)** | **2.9297**  **(1.6060)** | **12.63**  **(5.80)** |  | **22.5** | **84.9**  **(1.1)** | **0.9488**  **(0.4887)** | **4.06**  **(2.23)** |  | **74.0** | **85.1**  **(0.0)** | **0.6094**  **(0.2321)** | **2.51**  **(0.96)** |
|  | **222.6** | **1.875** | **7.5** |  | **29.5** | **221.3**  **(4.4)** | **3.0083**  **(1.7130)** | **13.46**  **(6.35)** |  | **68.0** | **222.6**  **(0.5)** | **1.9200**  **(0.9252)** | **8.36**  **(3.94)** |  | **98.0** | **222.6**  **(0.0)** | **1.7316**  **(0.4297)** | **7.21**  **(1.78)** |
| **2** | **401.4** | **0.625** | **2.5** |  | **7.5** | **402.5**  **(4.1)** | **2.8363**  **(1.5952)** | **11.60**  **(5.77)** |  | **22.0** | **401.8**  **(2.4)** | **0.8884**  **(0.5428)** | **3.89**  **(2.50)** |  | **81.0** | **401.4**  **(0.0)** | **0.5760**  **(0.2132)** | **2.38**  **(0.87)** |
|  | **438.8** | **1.875** | **7.5** |  | **29.0** | **438.8**  **(1.2)** | **3.0622**  **(1.3602)** | **14.07**  **(6.08)** |  | **65.5** | **438.8**  **(0.3)** | **1.8306**  **(0.7442)** | **7.97**  **(3.25)** |  | **96.5** | **438.8**  **(0.0)** | **1.8372**  **(0.4668)** | **7.63**  **(1.83)** |
| **3** | **601.6** | **3.750** | **15.0** |  | **59.0** | **601.5**  **(1.2)** | **4.0209**  **(2.0379)** | **18.05**  **(7.66)** |  | **88.0** | **601.6**  **(0.0)** | **3.5746**  **(1.4526)** | **15.67**  **(5.99)** |  | **100.0** | **601.6**  **(0.0)** | **3.6903**  **(0.6055)** | **15.41**  **(2.43)** |
| **8** | **1594.1** | **1.250** | **5.0** |  | **11.0** | **1594.4**  **(1.4)** | **2.2579**  **(0.9985)** | **11.09**  **(5.11)** |  | **47.0** | **1594.1**  **(0.0)** | **1.3772**  **(0.6279)** | **5.97**  **(2.76)** |  | **93.5** | **1594.1**  **(0.0)** | **1.2013**  **(0.3745)** | **4.99**  **(1.56)** |
|  | **1653.8** | **1.250** | **5.0** |  | **11.0** | **1654.6**  **(2.6)** | **2.3354**  **(1.3239)** | **10.69**  **(5.15)** |  | **47.0** | **1653.8**  **(0.3)** | **1.2715**  **(0.5704)** | **5.50**  **(2.53)** |  | **94.0** | **1653.8**  **(0.0)** | **1.1397**  **(0.3782)** | **4.73**  **(1.55)** |
| **9** | **1944.7** | **2.50** | **10.0** |  | **28.5** | **1945.1**  **(2.5)** | **3.0888**  **(1.7685)** | **13.88**  **(6.87)** |  | **77.0** | **1944.6**  **(0.6)** | **2.2476**  **(0.8507)** | **9.85**  **(3.52)** |  | **98.0** | **1944.7**  **(0.0)** | **2.3847**  **(0.5346)** | **9.88**  **(2.05)** |
| **10** | **2119.6** | **2.50** | **10.0** |  | **24.0** | **2119.6**  **(0.0)** | **3.2448**  **(1.4214)** | **14.42**  **(5.71)** |  | **79.0** | **2119.6**  **(0.0)** | **2.3855**  **(0.8959)** | **10.47**  **(3.69)** |  | **99.0** | **2119.6**  **(0.0)** | **2.4389**  **(0.5297)** | **10.16**  **(2.18)** |
|  | **2181.6** | **3.750** | **15.0** |  | **45.5** | **2181.5**  **(0.5)** | **3.5493**  **(1.8756)** | **16.04**  **(6.95)** |  | **94.5** | **2181.6**  **(0.0)** | **3.3398**  **(1.1033)** | **14.76**  **(4.41)** |  | **100.0** | **2181.6**  **(0.0)** | **3.6342**  **(0.6614)** | **15.14**  **(2.55)** |
