# Supplementary material for: Epistatic Association Mapping in Homozygous Crop Cultivars
Source: PLoS One. 2011 Mar 15;6(3):e17773. doi: 10.1371/journal.pone.0017773 (PMC3058038; doi:10.1371/journal.pone.0017773)
Supplement: Table S4 — Effect of the number of alleles on multi-QTL mapping in the third simulation experiment (200 replicates). (DOC) [file pone.0017773.s004.doc]

**Table S4. Effect of the number of alleles on multi-QTL mapping in the third simulation experiment (200 replicates)**

| **True value** | | | |  | **2** | | | |  | **3** | | | |  | **4** | | | |
| --- | --- | --- | --- | --- | --- | --- | --- | --- | --- | --- | --- | --- | --- | --- | --- | --- | --- | --- |
| **Chr.** | **Position**  **(cM)** | **Variance** | ***r*^2^ (%)** |  | **Power (%)** | **Position**  **(cM)** | **Variance** | ***r*^2^ (%)** |  | **Power (%)** | **Position**  **(cM)** | **Variance** | ***r*^2^ (%)** |  | **Power (%)** | **Position**  **(cM)** | **Variance** | ***r*^2^ (%)** |
| **1** | **85.1** | **0.625** | **2.5** |  | **78.5** | **85.1**  **(0.0)** | **0.6235**  **(0.2574)** | **2.55**  **(1.05)** |  | **34.0** | **85.0**  **(0.9)** | **0.7568**  **(0.3895)** | **3.36**  **(1.76)** |  | **30.5** | **85.0**  **(1.0)** | **0.7164**  **(0.3495)** | **3.39**  **(1.68)** |
|  | **222.6** | **1.875** | **7.5** |  | **98.5** | **222.6**  **(0.0)** | **1.8144**  **(0.4229)** | **7.44**  **(1.68)** |  | **78.0** | **222.6**  **(0.0)** | **1.6616**  **(0.7324)** | **7.25**  **(3.03)** |  | **81.5** | **222.6**  **(0.0)** | **1.4165**  **(0.7531)** | **6.71**  **(3.34)** |
| **2** | **401.4** | **0.625** | **2.5** |  | **83.0** | **401.4**  **(0.2)** | **0.6078**  **(0.2774)** | **2.49**  **(1.12)** |  | **31.0** | **401.7**  **(1.8)** | **0.7601**  **(0.4107)** | **3.36**  **(1.68)** |  | **34.5** | **401.5**  **(0.4)** | **0.5876**  **(0.2327)** | **2.85**  **(1.25)** |
|  | **438.8** | **1.875** | **7.5** |  | **99.5** | **438.8**  **(0.0)** | **1.8319**  **(0.4569)** | **7.52**  **(1.832)** |  | **83.5** | **438.9**  **(0.8)** | **1.7475**  **(0.7671)** | **7.74**  **(3.34)** |  | **83.0** | **438.8**  **(0.2)** | **1.4353**  **(0.6939)** | **6.79**  **(3.16)** |
| **3** | **601.6** | **3.750** | **15.0** |  | **100.0** | **601.6**  **(0.0)** | **3.6557**  **(0.6441)** | **15.00**  **(2.44)** |  | **98.0** | **601.6**  **(0.0)** | **3.4349**  **(1.0232)** | **15.19**  **(4.21)** |  | **98.0** | **601.6**  **(0.0)** | **2.7815**  **(1.0463)** | **13.22**  **(4.72)** |
| **8** | **1594.1** | **1.250** | **5.0** |  | **96.5** | **1594.1**  **(0.0)** | **1.1960**  **(0.3723)** | **4.89**  **(1.48)** |  | **52.0** | **1594.3**  **(1.7)** | **1.2030**  **(0.6128)** | **5.24**  **(2.64)** |  | **64.0** | **1594.3**  **(1.6)** | **0.9523**  **(0.4888)** | **4.50**  **(2.25)** |
|  | **1653.8** | **1.250** | **5.0** |  | **94.0** | **1653.8**  **(0.0)** | **1.2263**  **(0.3643)** | **5.00**  **(1.45)** |  | **64.5** | **1653.8**  **(0.8)** | **1.1963**  **(0.5555)** | **5.31**  **(2.50)** |  | **71.0** | **1653.8**  **(0.4)** | **1.0753**  **(0.5198)** | **5.06**  **(2.32)** |
| **9** | **1944.7** | **2.50** | **10.0** |  | **99.5** | **1944.7**  **(0.0)** | **2.4021**  **(0.4858)** | **9.86**  **(1.95)** |  | **83.5** | **1944.7**  **(0.4)** | **2.2861**  **(0.7854)** | **10.03**  **(3.22)** |  | **92.5** | **1944.7**  **(0.5)** | **1.8736**  **(0.8875)** | **8.83**  **(3.94)** |
| **10** | **2119.6** | **2.50** | **10.0** |  | **100.0** | **2119.6**  **(0.0)** | **2.4544**  **(0.4827)** | **10.07**  **(1.88)** |  | **80.5** | **2119.6**  **(0.0)** | **2.2437**  **(0.7762)** | **9.92**  **(3.44)** |  | **94.0** | **2119.6**  **(0.0)** | **1.8774**  **(0.9192)** | **8.86**  **(4.09)** |
|  | **2181.6** | **3.750** | **15.0** |  | **100.0** | **2181.6**  **(0.0)** | **3.6247**  **(0.6213)** | **14.86**  **(2.25)** |  | **94.5** | **2181.6**  **(0.0)** | **3.2162**  **(0.9805)** | **14.23**  **(4.08)** |  | **96.5** | **2181.6**  **(0.3)** | **2.9233**  **(1.0963)** | **13.77**  **(4.68)** |
