# Supplementary material for: Epistatic Association Mapping in Homozygous Crop Cultivars
Source: PLoS One. 2011 Mar 15;6(3):e17773. doi: 10.1371/journal.pone.0017773 (PMC3058038; doi:10.1371/journal.pone.0017773)
Supplement: Table S5 — Effect of allelic distribution on multi-QTL mapping in the fourth simulation experiment (200 replicates). (DOC) [file pone.0017773.s005.doc]

**Table S5. Effect of allelic distribution on multi-QTL mapping in the fourth simulation experiment (200 replicates)**

| **True value** | | | |  | **1:1** | | | |  | **1:2** | | | |  | **1:3** | | | |
| --- | --- | --- | --- | --- | --- | --- | --- | --- | --- | --- | --- | --- | --- | --- | --- | --- | --- | --- |
| **Chr.** | **Position**  **(cM)** | **Variance** | ***r*^2^ (%)** |  | **Power (%)** | **Position**  **(cM)** | **Variance** | ***r*^2^ (%)** |  | **Power (%)** | **Position**  **(cM)** | **Variance** | ***r*^2^ (%)** |  | **Power (%)** | **Position**  **(cM)** | **Variance** | ***r*^2^ (%)** |
| **1** | **85.1** | **0.625** | **2.5** |  | **81.5** | **85.1**  **(0.0)** | **0.6081**  **(0.2412)** | **2.50**  **(0.10)** |  | **61.5** | **85.0**  **(1.3)** | **0.7486**  **(0.4020)** | **2.93**  **(1.78)** |  | **43.5** | **84.6**  **(2.5)** | **0.8632**  **(0.4336)** | **3.03**  **(1.74)** |
|  | **222.6** | **1.875** | **7.5** |  | **98.0** | **222.6**  **(0.0)** | **1.7877**  **(0.4187)** | **7.37**  **(1.74)** |  | **91.5** | **222.6**  **(0.0)** | **2.1076**  **(0.7087)** | **8.15**  **(2.83)** |  | **75.0** | **222.5**  **(0.7)** | **2.4413**  **(0.9246)** | **8.83**  **(3.56)** |
| **2** | **401.4** | **0.625** | **2.5** |  | **84.0** | **401.4**  **(0.0)** | **0.6154**  **(0.2623)** | **2.52**  **(1.04)** |  | **58.0** | **401.6**  **(1.3)** | **0.6731**  **(0.2839)** | **2.59**  **(1.08)** |  | **45.5** | **401.4**  **(0.0)** | **0.7800**  **(0.4312)** | **2.87**  **(1.92)** |
|  | **438.8** | **1.875** | **7.5** |  | **96.5** | **438.8**  **(0.2)** | **1.8402**  **(0.3847)** | **7.57**  **(1.51)** |  | **89.0** | **438.8**  **(0.2)** | **2.0684**  **(0.5816)** | **7.96**  **(2.22)** |  | **79.0** | **438.8**  **(0.2)** | **2.4542**  **(0.9744)** | **8.91**  **(3.73)** |
| **3** | **601.6** | **3.750** | **15.0** |  | **100.0** | **601.6**  **(0.0)** | **3.6778**  **(0.6472)** | **15.15**  **(2.36)** |  | **95.5** | **601.5**  **(0.8)** | **4.1372**  **(0.8887)** | **15.95**  **(3.27)** |  | **87.0** | **601.5**  **(1.0)** | **4.8028**  **(1.4357)** | **17.19**  **(4.98)** |
| **8** | **1594.1** | **1.250** | **5.0** |  | **97.5** | **1594.1**  **(0.0)** | **1.2096**  **(0.3659)** | **4.98**  **(1.48)** |  | **82.5** | **1594.1**  **(0.0)** | **1.3370**  **(0.4607)** | **5.09**  **(1.65)** |  | **70.5** | **1594.2**  **(1.4)** | **1.5371**  **(0.6639)** | **5.61**  **(2.65)** |
|  | **1653.8** | **1.250** | **5.0** |  | **93.5** | **1653.8**  **(0.0)** | **1.2358**  **(0.3810)** | **5.10**  **(1.56)** |  | **84.5** | **1653.9**  **(0.7)** | **1.3868**  **(0.5021)** | **5.33**  **(1.94)** |  | **66.5** | **1653.9**  **(0.8)** | **1.5590**  **(0.6598)** | **5.57**  **(2.37)** |
| **9** | **1944.7** | **2.50** | **10.0** |  | **99.5** | **1944.7**  **(0.0)** | **2.4103**  **(0.5139)** | **9.92**  **(1.94)** |  | **93.0** | **1944.7**  **(0.0)** | **2.6935**  **(0.7144)** | **10.40**  **(2.75)** |  | **82.5** | **1944.7**  **(0.3)** | **3.2320**  **(0.8768)** | **11.75**  **(3.30)** |
| **10** | **2119.6** | **2.50** | **10.0** |  | **99.0** | **2119.6**  **(0.0)** | **2.4151**  **(0.5291)** | **9.94**  **(2.02)** |  | **95.0** | **2119.6**  **(0.0)** | **2.6748**  **(0.6496)** | **10.36**  **(2.47)** |  | **79.5** | **2119.6**  **(0.0)** | **3.1453**  **(0.9003)** | **11.42**  **(3.55)** |
|  | **2181.6** | **3.750** | **15.0** |  | **99.5** | **2181.6**  **(0.0)** | **3.6318**  **(0.6702)** | **14.97**  **(2.41)** |  | **95.0** | **2181.6**  **(0.0)** | **4.1479**  **(0.8240)** | **16.04**  **(2.97)** |  | **86.5** | **2181.6**  **(0.0)** | **4.8726**  **(1.5472)** | **17.36**  **(4.95)** |
